# Association of whole grains intake and the risk of digestive tract cancer: a systematic review and meta-analysis

**DOI:** 10.1186/s12937-020-00556-6

**Published:** 2020-06-03

**Authors:** Xiao-Feng Zhang, Xiao-Kai Wang, Yu-Jun Tang, Xiao-Xian Guan, Yao Guo, Jian-Ming Fan, Ling-Ling Cui

**Affiliations:** 1 Department of Nutrition and Food Hygiene, College of Public Health, Zhengzhou University, 100 Kexue Avenue, Zhengzhou, 450001 Henan China; 2 Nursing College of Henan University of Chinese Medicine, Zhengzhou, Henan China

**Keywords:** Whole grains, Digestive tract cancer, Colorectal cancer, Gastric cancer, Esophagus cancer, Meta-analysis

## Abstract

**Background:**

Several epidemiological studies have investigated the association between whole grains intake and digestive tract cancer risk; however, the results are still controversial. The purpose of this meta-analysis was to assess the association.

**Methods:**

Studies published before March 2020 were searched in database and other sources. The risk ratio (RR) with the 95% confidence interval (CI) were pooled using fix or random-effects models.

**Results:**

This meta-analysis included 34 articles reporting 35 studies, 18 studies of colorectal cancer, 11 studies of gastric cancer and 6 studies of esophagus cancer, involving 2,663,278 participants and 28,921 cases. Comparing the highest-intake participants with the lowest-intake participants for whole grains, we found that the intake of whole grains were inversely related to colorectal cancer (RR = 0.89, 95% CI: 0.84–0.93, *P* < 0.001), gastric cancer (RR = 0.64, 95% CI: 0.53–0.79, *P <* 0.001), esophagus cancer (RR = 0.54, 95% CI: 0.44–0.67, *P <* 0.001), respectively. However, subgroup analysis of colorectal cancer found no significant association in the case-control studies and studies of sample size < 500, and subgroup analysis of gastric cancer found no significant association in the cohort studies and studies of American population. No study significantly affected the findings in the sensitivity analysis. No publication bias was found in the studies for colorectal cancer and esophagus cancer except in the studies for gastric cancer.

**Conclusion:**

This meta-analysis provides further evidence that whole grains intake was associated with a reduced risk of digestive tract cancer. Our result supports the dietary guidelines that increase whole grains intake to reduce the risk of digestive tract cancer.

## Background

Globally, digestive tract cancer are common type of cancer. The global cancer statistics 2018 shows that the incidence of colorectal cancer, gastric cancer and esophageal cancer ranks third, fifth and seventh, and the mortality ranks second, third and sixth among all cancers, respectively [1]. Digestive tract cancer has become one of the major diseases that threaten human health. The occurrence of digestive tract cancer is related a variety of factors, of which approximately 5–10% can be attributed to genetic defects, whereas and the remaining 90–95% can be explained by unfavorable environment conditions or an unhealthy lifestyle [2, 3]. Studies have shown that diet plays an important role in the digestive tract cancer risk [4–6]. Grains are key components of the diet and supply much of the world’s energy and nutrient needs. They make up the largest proportion of recommended daily food intake [7, 8]. Due to their important role in most diets in the world, there was a lot of research on the relationship between grains consumption and health. With the development of grains research, the health function of whole grains food has been confirmed and aroused people’s interest [9]. Whole grains consist of the intact, ground, cracked, or flaked kernel after the removal of inedible parts. The principal anatomical components, the starchy endosperm, germ, and bran, are present in the same relative proportions as they exist in the intact kernel [10]. Compared to refined grains, whole grains are rich in dietary fiber and a variety of phytochemicals, which play an important role in preventing chronic diseases. Several studies have found a lower risk of obesity [11], cardiovascular disease [12, 13], type 2 diabetes [14], coronary heart disease [8, 15], stroke [8], cancer [13, 16] associated with a higher intake of whole grains.

A previous review of mostly case-control studies showed higher whole grains intake was associated with lower risk of several individual cancers, mainly of the digestive system [17], but limited data from cohort studies. Several epidemiological studies have investigated the relationship between whole grains intake and digestive tract cancer risk. However, these results are controversial. In 2003, Cullouh et al. reported that a statistically non-significant 17% increase in colon cancer risk was observed for women with the higher whole grains intakes [18]. However, in 2006, McCarl et al. report that that higher whole grains intake can reduce the risk of colorectal cancer by 19% for women [19]. In 2004, Lissowska et al. report that their study do not support a protective effect of whole grains for gastric cancer [20]. However, in 2002, Kasum et al. report that intake of whole grains was associated with reduced risk of upper aerodigestive tract cancers, including oropharyngeal, laryngeal, salivary, esophageal and gastric cancers [21]. From a public health perspective, it is important to clarify this issue. Therefore, the purpose of this meta-analysis was to determine whether there is an association between whole grains intake and digestive tract cancer.

## Methods

### Search strategy

Studies published before March 2020 were searched in database and other sources. In order to avoid missing any relevant research, we also searched the bibliography of the retrieved papers. The following keywords were used in the literature search: “grains” or “cereal” or “wheat” or “corn” or “rye” or “oats” or “oatmeal” or “bread” or “barley” or “bran” or “germ” or “colorectal cancer” or “colon cancer” or “rectal cancer” or “CRC” or “colorectal carcinoma” or “gastric cancer” or “stomach cancer” or “esophagus cancer” or “esophageal squamous cell carcinoma”. No restrictions were imposed.

### Study selection

Studies were considered for inclusion if they met the following criteria: (1) the research was a cohort study or a case–control study. (2) The research assessed the association between whole grains intake and the risk of colorectal cancer, esophageal cancer and gastric cancer. (3) The RR or odds ratios (OR) estimates with 95% confidence were reported or could be calculated. If data were duplicated in more than one study, the one with the largest number of cases or the longest follow-up period was included in the meta-analysis.

### Data extraction

Two independent researchers carried out an initial assessment of obtained literature to exclude those failing to meet the inclusion criteria. A further full-text assessment of the studies that had the potential to meet the criteria was made, and any disagreements were resolved by discussion between two authors or by the third investigator. Data extracted from each study included: the first author’s name, year of publication, country, cancer site, study design, diet assessment, simple (case), intake of whole grains, RR (OR) with 95%CI and variables adjusted.

### Quality assessment

The included case–control and cohort studies were assessed by two investigators using the scoring system of the Newcastle–Ottawa scale (NOS) [22]. The highest score was 9 points, and those with a score 7 were classified as high-quality literatures.

### Statistical analysis

Statistical analysis was performed using STATA version 12.0. The results were expressed as RR and 95% CI to measure the association between whole grains intake and the risk of digestive tract cancer. The heterogeneity assumption was examined by a Chi-square test based on a Q-test. Generally, *I*^*2*^ statistics of 25, 50, and 75% indicate low, moderate, and high levels of heterogeneity, respectively. If *p* < 0.05 and/or *I*^2^ > 50%, a random-effect model based was used to calculate pooled (RR) with the 95% confidence interval (CI). Otherwise, a fixed-effect model was used. Due to characteristics of participants, and adjustments for confounding factors were not consistent across studies, we further conducted several sensitivity and subgroup analyses to explore possible sources of heterogeneity and to examine the influence of various factors on the overall risk estimate. Sensitivity analysis was conducted by omitting one study each time and recalculating the pooled RR. Finally, we applied Begg’s method to assess bias.

## Results

### Literature search and study characteristics

**Fig. 1:**
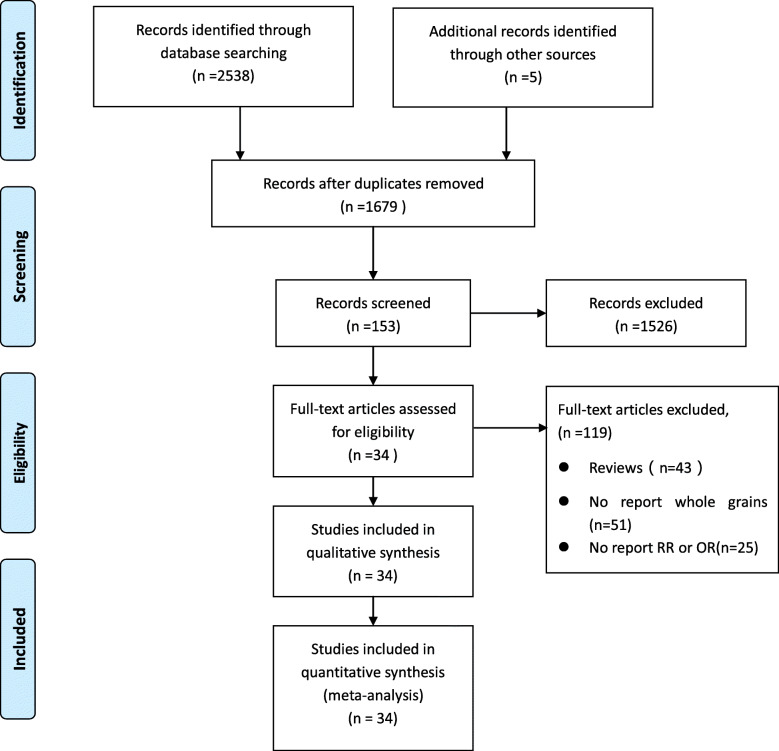
Flow chart of study selection. A flowchart of the research selection process of this meta-analysis is shown in Fig. 1. The search of database and other sources identified 1679 potentially relevant articles after duplicate exclusion. In addition, 1645 articles were excluded after further evaluations. Finally, 34 articles reporting 35 studies were selected for this meta-analysis

A flowchart of the research selection process of this meta-analysis is shown in Fig. 1. The search of database and other sources identified 1679 potentially relevant articles after duplicate exclusion. In addition, 1645 articles were excluded after further evaluations. Finally, 34 [18–21, 23–52] articles reporting 35 studies were selected for this meta-analysis.

Table 1 summarizes the general characteristics of these studies. All included studies were of high quality literature with scores greater than 7 points. Of the 35 studies, 14 were cohort studies and 22 were case-control studies, which included a total of 2663.278 participants and 28,921 cases. These studies were adjusted for a wide range of potential confounding factors, including age, sex, education, smoking, BMI, income, physical activity, energy intake, alcohol intake, red and processed meat intake etc.

**Table 1:** Characteristics of studies on whole grains intake and digestive tract cancers risk. (This table should be placed on line 132 in the sixth page)

Study	Country	Cancer site	Study design	Diet assessment	Simple (cases)	Intake comparison, High vs. low	RR/OR (95% CI)	Adjustment variables	Quality score
Ruth 1989 [23]	America	CRC	Case-control	Standard questionnaire	294 (147)	≥5 vs ≤1 servings/week	0.6 (0.4,1.1)	Age, education	7
Sandro 1994 [24]	Italy	CRC	Case-control	Standard questionnaire	264 (132)	Highest vs Lowest	1.03(0.54,1.95)	Age, sex, education, smoking, modification of diet in the past.	7
Martha 1997 [25]	America	CRC	Case-control	Frequency questionnaire	4403 (1993)	> 1.9 vs < 0.5 servings/day	1.0 (0.8,1.4)	Age, body mass index, physical activity, use of aspirin/NSAID, presence or absence of a first-degree relative with colorectal cancer, total energy intake and calcium	7
Cullouh 2003 [18]	America	CRC	Cohort	Frequency questionnaire, 68 items	133,163 (508)	≥11.0 vs < 2.0 servings/week ≥11.2 vs < 2.5 servings/week	Man 0.95 (0.64,1.42) Woman 1.17 (0.73,1.87)	Age, exercise metabolic equivalent of tasks, aspirin, smoking, family history of colorectal cancer, body mass index, education, energy	7
Wu 2004 [26]	America	CRC	Cohort	Frequency questionnaire, 131 food items	51,129 (561)	Highest vs Lowest	0.75 (0.57,1.00)	Age, family history of colorectal cancer in first degree relative, history of endoscopy, physical activity, pack years of smoking before age 30, race, aspirin use, energy	7
Larsson 2005 [27]	Swedish	CRC	Cohort	Frequency Questionnaire 67 food items	61,433 (805)	≥4.5 vs < 1.5 servings/day	0.80 (0.60,1.06)	Age, body mass index, education, energy, saturated fat, calcium, red meat, fruits and vegetables	7
Carl 2006 [19]	America	CRC	Cohort	Frequency questionnaire 127 food items	35,197 (757)	≥19 *v* ≤ 3.5 servings/week	0.81 (0.66,0.99)	Age	7
Schatzkin 2007 [28]	America	CRC	Cohort	Frequency questionnaire 124 food items	567,169 (2974)	1.3 vs 0.2 servings /day	0.79 (0.70,0.89)	Age, sex, physical activity, smoking, HRT (women), red meat, dietary calcium, dietary folate, energy	8
Christin 2009 [29]	America	CRC	Case-control	Frequency questionnaire 124 food items	1904 (945)	2.8 vs 16.4 servings/week 2.9 vs 18.9 servings/week	Whites 0.93 (0.66,1.31) African-Americans 0.67 (0.21,1.42)	Adjusted for age, sex, education, income, BMI 1 year ago, physical activity, family history, nonsteroidal anti-inflammatory drug use, and total energy intake.	7
Egeberg 2010 [30]	Denmark	CRC	Cohort	Frequency Questionnaire 192 food items	160,725 (744)	> 160 vs ≤75 g/day	Colon cancer, men 0.61 (0.43,0.86) Rectal cancer, men 0.88 (0.57,1.36) Colon cancer, women 0.92 (0.63,1.35) Rectal cancer, women 0.81 (0.50,1.30)	Age, body mass index, alcohol intake, school education, red and processed meat, HRT (women), leisure time physical activity	7
Fung 2010 [31]	America	CRC	Cohort	Frequency questionnaire 140 food items	132,746 (2464)	High vs Low	Men 0.94 (0.88,0.99) Women 0.95 (0.89,1.02)	Age, body mass index, alcohol, family history of colorectal cancer, colonoscopy, history of polyps,	8
Kyro 2013 [32]	Denmark	CRC	Cohort	Frequency questionnaire	10,800 (1123)	Men > 71 vs < 31 Women > 68 vs < 30 g/day	0.86(0.69,1.06)	Alcohol intake, smoking status, education, intake of red and processed meat, BMI, and energy intake	7
Suhad 2015 [33]	Jordan	CRC	Case-control	Frequency questionnaire 109 food items	407 (167)	1/4 the time vs All the time	0.32(0.12,0.84)	Odds ratios, 95% confidence intervals, and tests for trend for CRC by weekly consumption	8
Reema 2016 [34]	Jordan	CRC	Case-control	Frequency questionnaire	501 (220)	≥3/4 vs < 1/4 of the Meals	0.44 (0.22,0.92)	Adjusted for age, gender, total energy, red meat consumption, physical activity, smoke	9
Sandro 2016 [35]	Brazil	CRC	Case-control	Frequency questionnaire	270 (169)	1.2 vs 4.1 serving/month	0.96(0.92,1.01)	Different types of food	7
Bakken 2016 [36]	Norway	CRC	Cohort	Frequency questionnaire	78,254 (795)	> 34 vs < 180 g/day	0.89 (0.72,1.09)	Age as the time scale and adjusted for body mass index, hormone replacement therapy, smoking, alcohol consumption,	7
Caroline 2019 [37]	America	CRC	Cohort	Frequency questionnaire 68 food items	112,149 (1742)	Man 9.2 vs 174 g/day Woman 11 vs 168 g/day	0.92 (0.79, 1.08)	Age, sex, total energy intake, body mass index, smoking status, physical activity, hormone replacement therapy use, total calcium red and processed meat,	8
Xiaosheng 2019 [38]	America	CRC	Cohort	Frequencyquestionnaire	138,773 (3178)	Men 4.00 vs 58.3 Women 3.18 vs 39.1 g/day	Men 0.73 (0.55,0.96) Women 1.08 (0.84,1.38)	Age, family history of colorectal cancer, history of lower gastrointestinal endoscopy, smoking status, body mass index physical activity, alcohol intake, regular aspirin use, regular multivitamin use, calcium intake, vitamin D intake	8
Vecchia 1988 [39]	Italy	GC	Case-control	Structured questionnaire	1819 (206)	High vs low	0.40(0.16,0.98)	Age, geographic area, sex, education	8
Anna 1990 [40]	America	GC	Case-control	Structured questionnaire	274 (137)	High vs low	0.42(0.24,0.74)		7
Boeing 1991 [41]	Poland	GC	Case-control	Frequency questionnaire 43 food items	1482 (741)	High vs Low	0.62(0.47,0.82)	Age, sex, occupation, education, and residency sex, occupation, education, and residency	7
Boeing 1991 [42]	Germany	GC	Case-control	Interviewer administered questionnaire	722 (143)	Highest vs Lowest	0.37(0.22,0.62)	Age, sex, and hospital	8
Jedrychowski 1992 [43]	Poland	GC	Case-control	Structured questionnaire	1482 (741)	Highest vs Lowest	0.18(0.07,0.44)	Age, sex, education, occupation of the index person and residency	7
Hansson 1993 [44]	Sweden	GC	Case-control	Structured questionnaire	1135 (456)	High vs Low	0.89(0.79,1.01)	Age, gender, SES and consumption of a food item during adolescence and 20 years prior to interview	7
Sonia 1997 [45]	Italy	GC	Case-control	Structured questionnaire	2746 (722)	High vs Low	0.63(0.28,1.40)	Age, sex, area of residence and education	8
Liliane 1999 [46]	Italy	GC	Case-control	Structured questionnaire	6862 (3336)	High vs Low	0.5 (0.4,0.7)	Age and sex.	7
Marjorie 2001 [47]	America	GC	Cohort	Frequency questionnaire	970,045 (1349)	> 4 vs < 1 sum of days/week	Man0.90(0.77,1.06) Woman0.97(0.77,1.24)	Age, education, smoking, BMI, multivitamin and vitamin C use, aspirin use, race, and family history	7
Kasum 2002 [21]	America	GC	Cohort	Frequency questionnaire	34,651 (169)	6.9–12.5 vs 13.0–108.5 servings/week	0.61 (0.34,0.81)	Age and energy intake	8
Lissowska 2004 [22]	Poland	GC	Case-control	Frequency questionnaire	737 (274)	High vs Low	1.05(0.65,1.69)	Age, sex, education, smoking, and calories from food	7
Levi 2000 [48]	Switzerland	EC	Case-control	Frequency questionnaire	450 (101)	> 10 vs < 4 times/week	0.3(0.1–0.6)	Age, sex, education, smoking habits, and vegetables, fruits, alcohol and energy intake	7
Honglei 2002 [49]	America	EC	Case-control	Health Habits Questionnaire	697 (124)	High vs Low	0.25 (0.12, 0.52)	Age, sex, energy intake, respondent type, BMI, alcohol use, tobacco use, education, family history	8
Kasum 2002 [21]	America	EC	Cohort	Frequency questionnaire	34,651 (169)	> 21 vs < 3 servings/week	0.53(0.34–0.81)	Age, pack-years of smoking, alcohol use and energy intake.	8
Mahsa 2012 [50]	Iran	ESCC	Case-control	Frequency questionnaire	153 (47)	0.25 vs 0.03 Serving/day	0.57(0.28–0.76)	Age, sex, total energy intake, gastroesophageal reflux disease symptoms, medication use, BMI, smoking, physical activity	9
Sewram 2014 [51]	South Africa	EC	Case-control	Structured questionnaire	1858 (670)	> 5 vs > 1 per week	Men0.66(0.40–1.10) Woman0.78(0.45,1.34)	Age, hospital, residence, and years of education.	7
Skeie 2016 [52]	Norway,	EC	Cohort	Frequency questionnaire	113,933 (112)	> 160 vs < 62 g/day	0.55 (0.31–0.97)	Age, sex, education, smoking	7

**Abbreviations:***RR* Relative risk, *CI* Confidence interval, *ORs* Odds ratios, *CRC* Colorectal cancer, *GC* Gastric Cancer, *EC* Esophageal, *ESCC* Esophageal Squamous Cell Cance

### Whole grains intake and overall digestive tract cancer risk

**Fig. 2:**
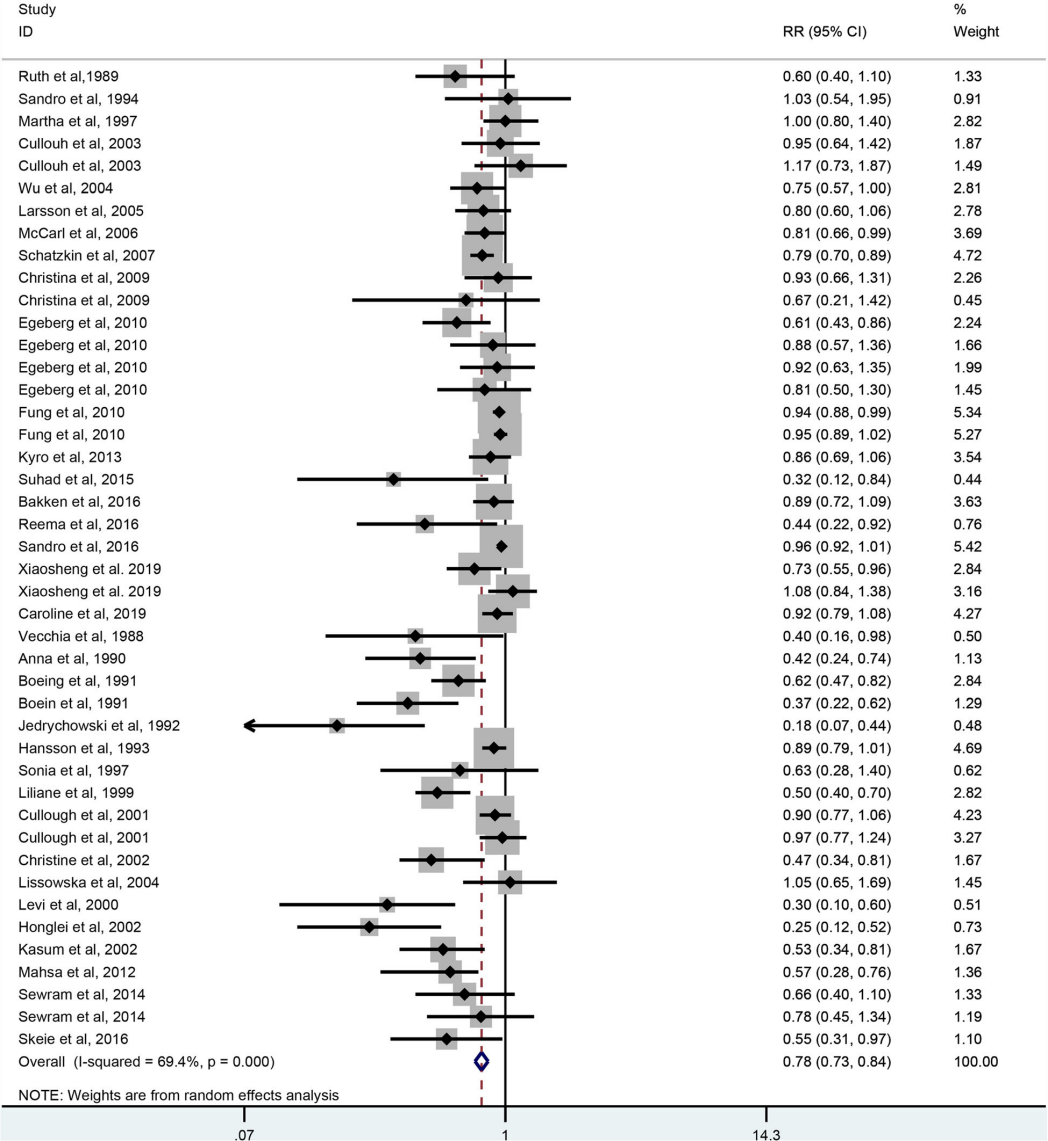
The forest plot of whole grains intake and digestive tract cancers risk. As shown in Fig. 2, thirty-five studies were included in the analysis of whole grains intake and digestive tract cancer risk. The result showed that whole grains consumption can reduce the risk of digestive tract cancer by 22% (RR = 0.78, 95% CI: 0.73–0.84, *P <* 0.001), with a significant heterogeneity (*I*^*2*^ = 69.4%, *P <* 0.001)

As shown in Fig. 2, 35 studies including 266,378 participants and 28,921 case. Were selected for the analysis of whole grains intake and digestive tract cancer risk. The result showed that whole grains consumption can reduce the risk of digestive tract cancer by 22% (RR = 0.78, 95% CI: 0.73–0.84, *P <* 0.001), with a significant heterogeneity (*I*^*2*^ = 69.4%, *P <* 0.001).

### Whole grains intake and colorectal cancer risk

**Fig. 3:**
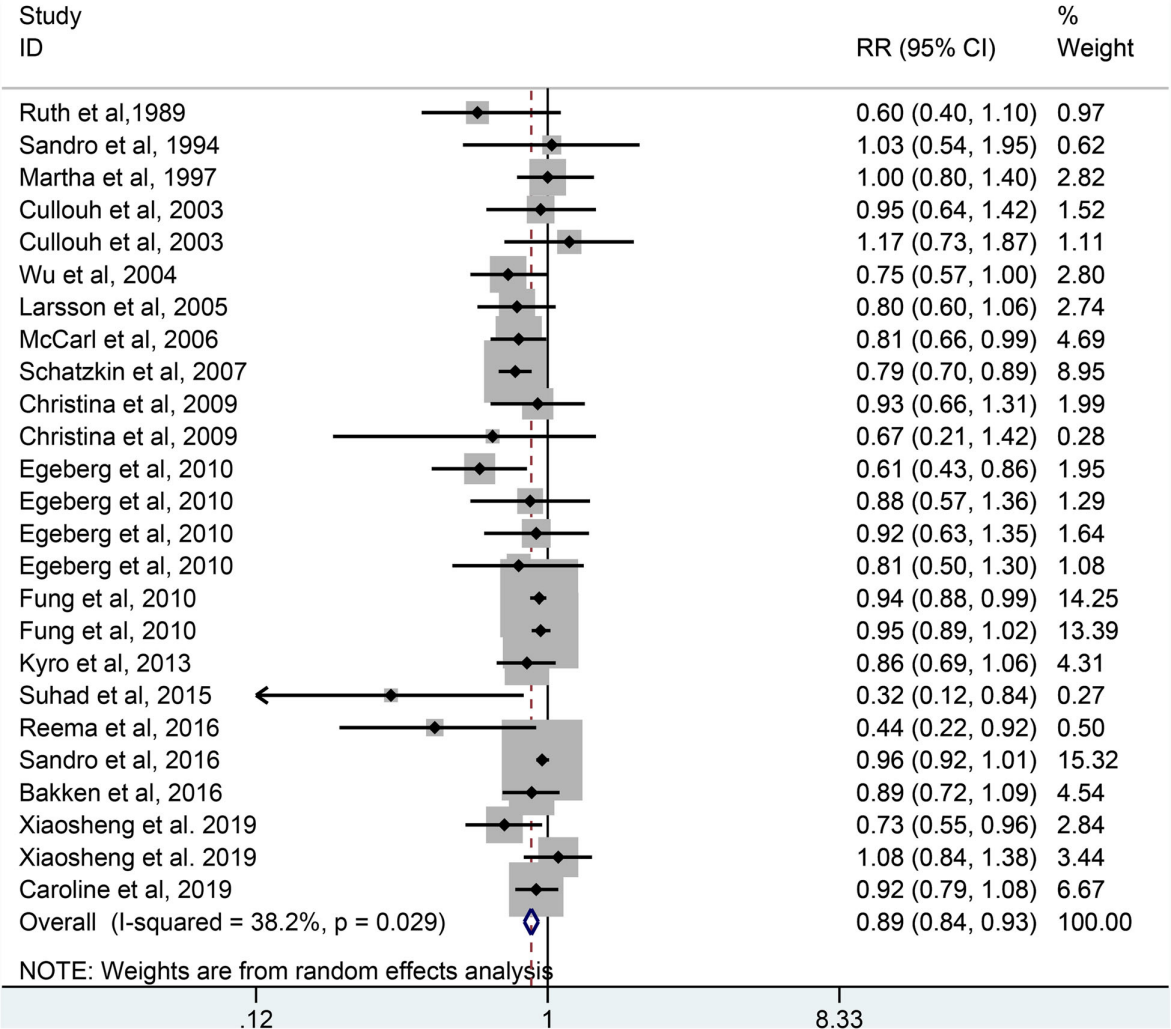
The forest plot of whole grains intake and colorectal risk. As shown in Fig. 3, eighteen studies were included in the analysis of whole grains intake and colorectal cancer risk. The result showed that whole grains consumption reduced the risk of colorectal cancer by 11% (RR = 0.89, 95% CI: 0.84–0.93, *P <* 0.001), with a slight heterogeneity (*I*^*2*^ = 38.2%, *P =* 0.029)

As shown in Fig. 3, 18 studies including 1,489,581 participants and 19,424 case were selected for the analysis of whole grains intake and colorectal cancer risk. The result showed that whole grains consumption reduced the risk of colorectal cancer by 11% (RR = 0.89, 95% CI: 0.84–0.93, *P <* 0.001), with a slight heterogeneity (*I*^*2*^ = 38.2%, *P =* 0.029).

There is a slight heterogeneity existed across the studies of whole grains intake and colorectal cancer risk and subgroup analysis were performed to find the source of heterogeneity. As shown in Table 2, the subgroup analysis was conducted according to the study design, sex, geographic location, publication year, sample size and whether adjust for energy intake. The result indicated that whole grains intake was protective factor for the studies of sample size ≥500 (RR: 0.91, 95% CI: 0.88–0.94, *P <* 0.001), but no significant association was found in the studies of sample size < 500 (RR: 0.76, 95% CI: 0.51–1.12, *P =* 0.170). In the subgroup analysis of sex, geographic location, publication year and whether adjust for energy intake, no statistically significant heterogeneity was found in the studies of women (*I*^*2*^ = 0%, *P =* 0.619), studies of Europe (*I*^*2*^ = 0%, *P =* 0.732), studies of publication year before 2010 (*I*^*2*^ = 0%, *P =* 0.622), studies of adjustment for energy (*I*^*2*^ = 4.6%, *P =* 0.399).

**Table 2:** Subgroup analysis of whole grains intake and risk of colorectal cancer

Subgroups	No. of studies	No. of Participants (Cases)	RR (95% CI)	***P***	Heterogeneity Test		
					Chi-Square	***I***^***2***^	***p***_***het***_
**All studies**	18	1,489,581(19424)	0.89(0.84,0.93)	< 0.001	38.82	38%	0.029
**Study design**							
Cohort	11	1,481,538(15519)	0.91(0.88,0.94)	< 0.001	22.95	30.3%	0.115
Case-control	7	8043(3905)	0.95(0.91,1.00)	0.030	13.33	47.5%	0.064
**Sex**							
Men	7	236,055(4826)	0.80(0.69,0.92)	0.001	14.07	50.3%	0.050
Women	8	454,822(7126)	0.94(0.89,0.99)	< 0.001	6.25	0%	0.619
**Geographic locations**							
Europe	5	311,476(3599)	0.84(0.75,0.93)	0.001	4.40	0%	0.732
America	10	1,176,927(15769)	0.92(0.88,0.95)	< 0.001	19.83	34.5%	0.099
**Publication year**							
Before 2010	9	854,956(8822)	0.82(0.76,0.89)	< 0.001	8.07	0%	0.622
After 2010	9	634,625(10602)	0.94(0.91,0.97)	< 0.001	21.73	40.2%	0.060
**Sample size**							
≥ 500	14	1,488,346(18809)	0.91(0.88,0.94)	< 0.001	27.76	27.9%	0.115
< 500	4	1235(615)	0.76(0.51,1.12)	0.170	8.20	63.4%	0.042
**Adjustment for energy**							
Yes	9	881,482(10198)	0.85(0.79,0.92)	< 0.001	10.48	4.6%	0.399
No	9	608,299(9226)	0.94(0.91,0.97)	< 0.001	21.84	40.5%	0.058

### Whole grains intake and gastric cancer risk

**Fig. 4:**
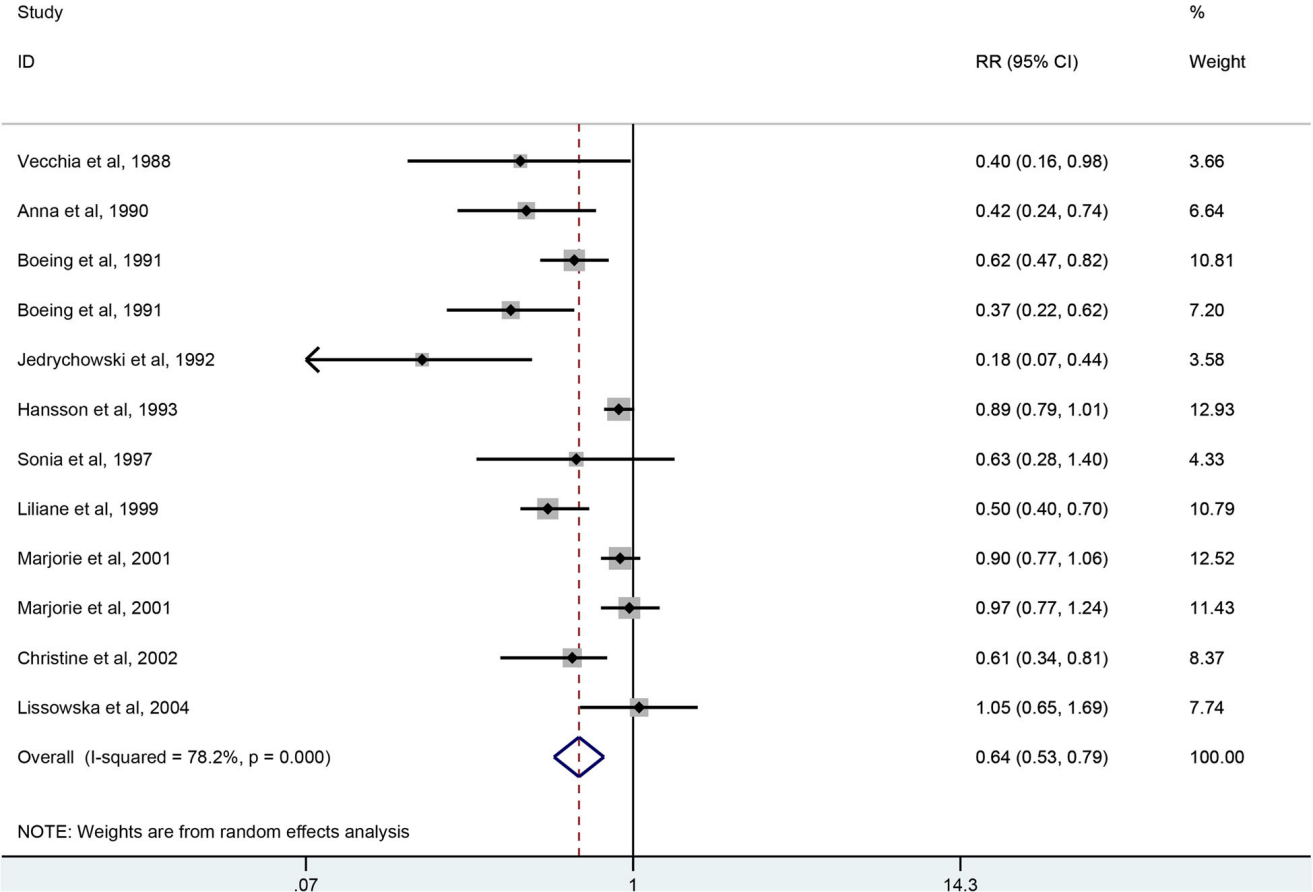
The forest plot of whole grains intake and gastric risk. As shown in Fig. 4, eleven studies were included in the analysis whole grains intake and gastric cancer risk. The result showed that whole grains consumption reduced the risk of gastric cancer by 36% (RR = 0.64, 95% CI: 0.53–0.79, *P <* 0.001), with a significant heterogeneity (*I*^*2*^ = 78.2%, *P =* 0.001)

As shown in Fig. 4, 11 studies including 1,021,955 participants and 8274 case were selected for the analysis of whole grains intake and gastric cancer risk. The result showed that whole grains consumption reduced the risk of gastric cancer by 36% (RR = 0.64, 95% CI: 0.53–0.79, *P <* 0.001), with a significant heterogeneity (*I*^*2*^ = 78.2%, *P =* 0.001).

There is a significant heterogeneity existed across the studies of whole grains intake and gastric cancer risk and subgroup analysis were performed to find the source of heterogeneity. As shown in Table 3, the subgroup analysis was conducted according to the study design, geographic location, sample size,and whether adjustment for energy. The result indicate that whole grains intake was protective factor for case-control studies (RR = 0.55, 95% CI: 0.41–0.74, *P <* 0.001) and studies of Europe (RR = 0.64, 95% CI: 0.53–0.79, *P <* 0.001), but no significant association was found in cohort studies (RR = 0.89, 95% CI: 0.78–1.01, *P =* 0.070) and studies of America (RR = 0.70, 95% CI: 0.50–1.00, *P =* 0.051). In the subgroup analysis of study design, heterogeneity decreased significantly in the cohort studies (*I*^*2*^ = 41.7%, *P =* 0.180). However, there was a significant heterogeneity in the case-control studies (*I*^*2*^ = 80.8%, *P* < 0.001).

**Table 3:** Subgroup analysis of whole grains intake and risk of gastric cancer

Subgroups	No. of studies	No. of Participants (Cases)	RR (95% CI)	***P***	Heterogeneity Test		
					Chi-Square	***I***^***2***^	***p***_***het***_
**All studies**	11	1,021,955(8274)	0.64(0.53,0.79)	< 0.001	50.42	78.2%	< 0.001
**Study design**							
Cohort	2	1,004,696(1518)	0.89(0.78,1.01)	0.070	3.43	41.7%	0.180
Case-control	9	17,259(6756)	0.55(0.41,0.74)	< 0.001	41.59	80.8%	< 0.001
**Geographic locations**							
Europe	8	16,985(6619)	0.64(0.53,0.79)	< 0.001	50.42	78.2%	< 0.001
America	3	1,004,970(1655)	0.70(0.50,1.00)	0.051	9.92	69.8%	0.019
**Sample size**							
≥ 500	10	1,021,681(8137)	0.67(0.54,0.82)	< 0.001	45.51	78.0%	< 0.001
< 500	1	274(137)	0.42(0.24,0.74)	0.003	N/A	N/A	N/A
**Adjustment for energy**							
Yes	1	34,651(169)	0.61(0.34,0.81)	0.026	N/A	N/A	N/A
No	10	987,204(8105)	0.65(0.52,0.80)	< 0.001	49.03	79.6%	< 0.001

**Abbreviations:***N/A* Not applicable

### Whole grains intake and esophagus cancer risk

**Fig. 5:**
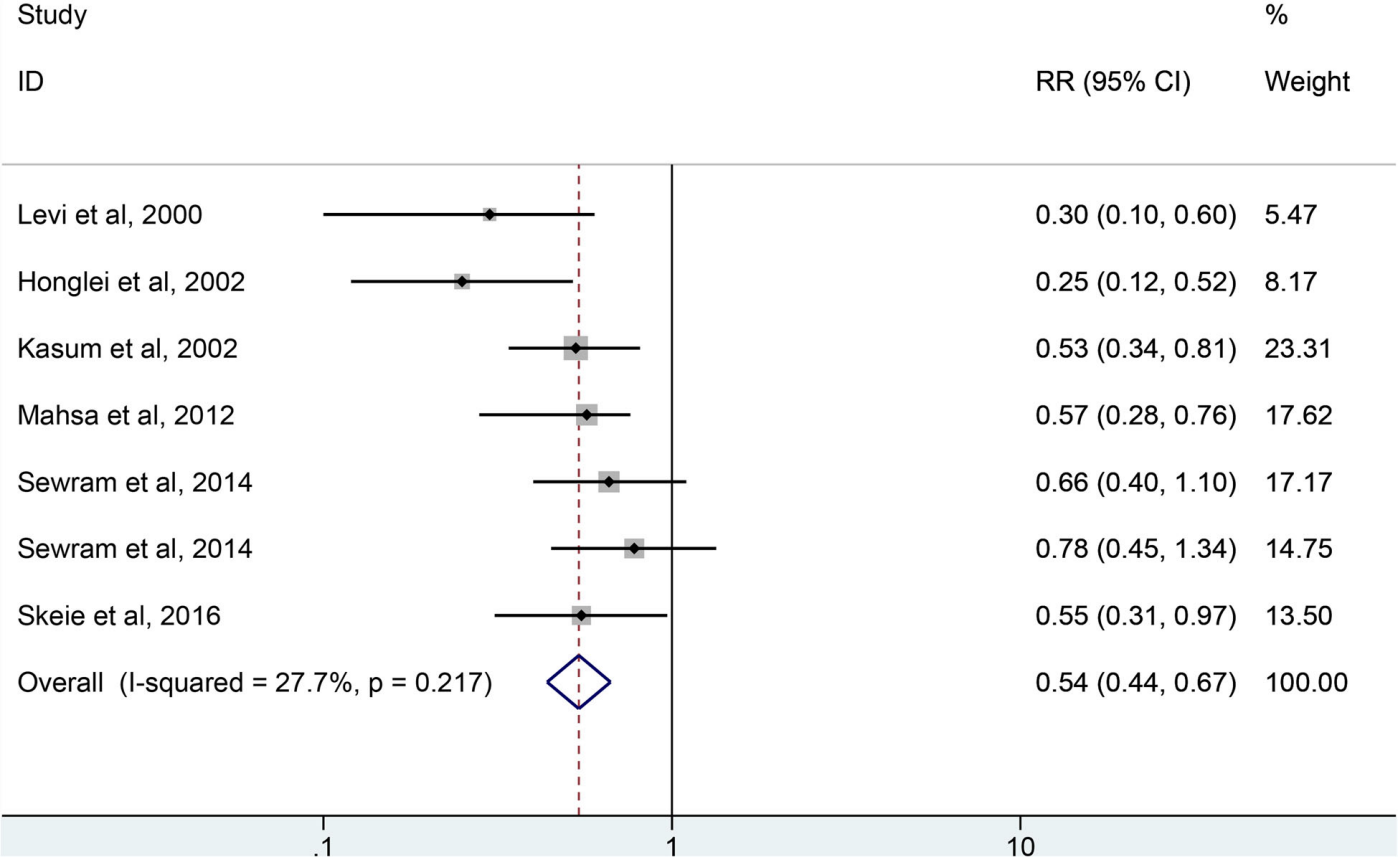
The forest plot of whole grains intake and esophagus risk. As shown in Fig. 5, six studies were included in the analysis of whole grains intake and esophagus cancer risk. The result showed that whole grains consumption reduced the risk of esophagus cancer by 47% (RR = 0.54, 95% CI: 0.44–0.67, *P <* 0.001) with no heterogeneity (*I*^*2*^ = 27.7%, *P =* 0.217)

As shown in Fig. 5, 6 studies including 151,742 participants and 1223 case were selected for the analysis of whole grains intake and esophagus cancer risk. The result showed that whole grains consumption reduced the risk of esophagus cancer by 47% (RR = 0.54, 95% CI: 0.44–0.67, *P <* 0.001) with no statistically significant heterogeneity (*I*^*2*^ = 27.7%, *P =* 0.217).

### Sensitivity analysis and publication bias

Sensitivity analysis suggested that no individual study significantly affected the pooled RR, which indicated that our results were statistically robust. The Begger test indicated no publication bias was found in the studies for colorectal cancer and esophagus cancer except in the studies for gastric cancer.

## Discussion

In this systematic review and meta-analysis, we evaluated the association between whole grains intake and the risk of digestive tract cancer. The results suggest that higher intake of whole grains were associated with lower risk of colorectal cancer, gastric cancer and esophageal cancer.

Previous studies have reported the association between whole grains and digestive tract cancer risk. In 1998 Liliane et al. [17]. first reported the association between whole grains and digestive tract cancer, and the result shows that higher intake of whole grains can reduce the risk of cancer in the colorectal cancer, gastric cancer and esophageal cancer. However, the literatures included in this study are almost case-control studies with limited sample size. In 2011 Dagfinn Aune et al. [16]. reported that a high intake of whole grains was associated with a decreased risk of colorectal cancer. In 2017, A. R. Vieira et al. [6]. reported that colorectal cancer risk decrease in 17% for each 90 g/day increase of whole grains. In 2018, Yujie Xu et al. [53]. reported that whole grains consumption was associated with decreased gastric risk. In 2019, Tonghua Wang et al. reported that whole grains consumption can reduce 13% risk of gastric cancer [54]. In 2018,Rachna Khosla et al. [55]. reported that the association between whole-grains foods and decreased esophageal cancer risk has been seen.

Whole grains may influence cancer risk through a variety of mechanisms. First, whole grains are rich in a variety of phytochemicals, and these bioactive components offer potential benefits in reducing cancer [56–58]. Second, whole grains are an important source of dietary fiber. Dietary fiber can increase the volume of feces and shorten the transit time of the intestines, thereby diluting carcinogens and reducing their absorption in the intestinal epithelium. Dietary fiber can also be fermented in the colon into short chain fatty acids including butyrate. Butyrate is the fuel of choice for mucosal cells and has the potential to promote apoptosis and anti-tumor, thereby reducing tumor growth. They also lower the intestinal pH, thereby reducing the solubility of free bile acids and reducing their carcinogenic activity. In addition, dietary fiber can remove nitrite in the stomach and reduce the concentration of nitroso compounds under strong acid conditions. Nitrate will increase the risk of gastric cancer [16, 59, 60]. Third, Consumption of whole grains has been proven to reduce the risk of obesity and improve metabolic disorders, and it can reduce risk of cancer [59, 61–64]. Fourth, whole grains have antioxidant and anti-inflammatory properties and it can improves blood sugar response and reduces insulin resistance, thereby reducing the risk of cancer [65–68].

Due to the difference of pathological location and etiology between colorectal, gastric and esophageal cancers, we did not conduct the subgroup analysis, sensitivity analysis and publication bias of whole grains intake and overall digestive tract cancer risk. In the meta-analysis of whole grains intake and colorectal cancer risk, we found a slight heterogeneity, and subgroup analysis was performed to find the source of heterogeneity. When subgroup analysis based on sex, geographic location, publication year and whether adjust for energy intake, no statistically significant heterogeneity was found in the studies of women, studies of Europe, studies of publication year before 2010 and studies of adjustment for energy; suggesting that sex, geographic location, publication year and whether adjust for energy intake may be a potential source of heterogeneity. In the meta-analysis of gastric cancer, there is significant heterogeneity. When subgroup analysis based on the study design showed that the heterogeneity was not significant in the cohort study,but the heterogeneity was still significant in the case-control study. This may be due to the recall bias and selection bias in case-control studies. In addition, the number of cohort studies is limited. Therefore, more cohort studies are needed to adequately adjust for potential confounders. Due to the significant publication bias of whole grains intake and gastric cancer risk, the association of whole grains and gastric cancer should be more cautious to interpret. In addition, we did not perform the subgroup analysis of whole grains and esophagus cancer risk because there was no statistically significant heterogeneitys.

There were limitations to our meta-analysis that should be considered. First, this study lacks high quality epidemiological studies. Due to the differences in methods for assessing whole-grains intake, we are unable to perform a meta-analysis of dose-response. Second, differences in the definitions of whole grains and in the categories of whole grains foods among studies might also be another possible source of heterogeneity. Third, there was high heterogeneity and publication bias in the analysis of whole grains and gastric. The existence of heterogeneity and publication bias makes it more cautious to interpret the results of this meta-analysis. Fourth, the included studies are mainly from Europe and America, lacking research in other regions. Finally, only published studies were included in the meta-analysis, the limitation of possible publication bias should be taken into consideration.

## Conclusion

In conclusion, intake of higher whole grains can reduce the risk of colorectal cancer, gastric cancer and esophageal cancer. However, it should be more cautious to interpret the association of whole grains and gastric cancer because there is a high heterogeneity and significant publication bias. More high-quality study is needed in the future to clarify dose-response relationships and to assess the relationship between whole grains and digestive tract cancer.

## Data Availability

The tables and figures supporting the conclusions of this article are included within the article.

## Abbreviations

RR: Relative risk
CI: Confidence interval
ORs: Odds ratios
NOS: Newcastle Ottawa Scale
CRC: Colorectal cancer
GC: Gastric Cancer
EC: Esophageal
ESCC: Esophageal Squamous Cell Cancer
